# Identifying childhood correlates of adult purpose and meaning across 22 countries (Global Flourishing Study)

**DOI:** 10.1038/s44184-025-00127-9

**Published:** 2025-04-30

**Authors:** Eric S. Kim, Matt Bradshaw, R. Noah Padgett, Ying Chen, Koichiro Shiba, James L. Ritchie-Dunham, Brendan W. Case, Byron R. Johnson, Tyler J. VanderWeele

**Affiliations:** 1 Department of Psychology, University of British Columbia, Vancouver, Canada; 2 Human Flourishing Program, Institute for Quantitative Social Science, Harvard University, Cambridge, MA USA; 3 Lee Kum Sheung Center for Health and Happiness, Harvard T.H. Chan School of Public Health, Boston, MA USA; 4 Institute for Studies of Religion, Baylor University, Waco, TX USA; 5 Department of Epidemiology, Harvard T.H. Chan School of Public Health, Boston, MA USA; 6 Department of Epidemiology, Boston University School of Public Health, Boston, MA USA; 7 Rosenthal Department of Management, McCombs School of Business, University of Texas at Austin, Austin, TX USA; 8 School of Public Policy, Pepperdine University, Malibu, CA USA; 9 Department of Biostatistics, Harvard T.H. Chan School of Public Health, Boston, MA USA

**Keywords:** Psychology, Epidemiology

## Abstract

How might we cultivate a life imbued with purpose and meaning? Though common experience indicates purpose and meaning are inherently important, empirical evidence confirms they are also linked to improved health/well-being. However, childhood antecedents of purpose and meaning in adulthood are understudied. We analyzed nationally representative data from 22 countries in the Global Flourishing Study (*N* = 202,898) and evaluated whether 11 aspects of a child’s upbringing correlate with purpose and meaning in adulthood, and if these associations vary by country. Some childhood factors were associated with increased purpose and meaning, including: good health, good relationship with parents, economic stability, regular religious service attendance, being female, and older birth cohorts. Childhood factors associated with decreased purpose and meaning included: abuse, feeling alienated, poor health, economic hardship, and loss of a parent. These findings may inform the development of programs designed to foster purpose and meaning that are globally adaptable and locally nuanced.

## Introduction

How might we cultivate a life imbued with purpose and meaning? An emerging body of research is beginning to shed light on this question. However, most existing studies have examined this question only within a single country. Conducting cross-national research offers a rare opportunity to enhance our understanding of how contrasting sociopolitical and economic environments shape purpose and meaning. Assessing how various childhood correlates influence adult purpose and meaning across countries with different national contexts can help us understand which correlates consistently shape purpose and meaning globally, and which are amplified (or dampened) by the specific contexts of individual countries. To date, these questions have arguably been underexplored. Addressing these knowledge gaps can enrich our understanding of the developmental roots of purpose and meaning, and also highlight potential areas for early intervention, including universal approaches as well as culturally specific adaptations.

A growing consensus in the psychological literature suggests that meaning can be understood through a tripartite framework, encompassing three core components: (1) *motivational purpose* (the extent that people have clear, overarching life aims that align with one’s values and helps prioritize decisions and actions), (2) *cognitive coherence/comprehension* (the intellectual perception that one’s life, values, and relations to the world form an intelligible pattern within a broader context or narrative), and (3) *affective significance/mattering* (the emotional value or importance one places on life experiences)^1–3^. The focus of our study is on purpose and meaning, and they are both central components of well-being^2,4–16^. These factors are important in their own right, but also important because they shape people’s trajectories of psychological-, social-, behavioral-, spiritual-, and physical-well-being. Viktor Frankl suggested that a higher purpose (and meaning) leads to a greater will to live^5^, which motivates people to endure short-term discomfort for the sake of longer-term rewards. Building on this theory a relatively recent review paper^13^, synthesized evidence showing that people with a higher sense of purpose and meaning experience reduced stress, recover more quickly when perturbed by negative stimuli, display a heightened ability to curb impulsivity, report higher self-efficacy, and use healthier coping methods^13,17–22^.

Thus, people with a heightened sense of purpose and meaning are better equipped to manage stress and recover more quickly from setbacks. This resilience often leads people to resist impulsively indulging in unhealthy behaviors such as smoking, overeating comfort foods, or excessive drinking^13^. Instead, their strong self-efficacy and effective coping strategies tend to guide them towards healthier choices, such as consuming more nutritious diets and engaging in physical activity as well as preventive healthcare services^13^. In line with this theory, accumulating research demonstrates that a higher sense of purpose and meaning is associated with better health behaviors including healthier/increased use of: preventive healthcare services, physical activity, diet, sleep and reduced drug misuse^23–28^. Additionally, such people often show improved biological functioning marked by reduced epigenetic age, allostatic load, and inflammation^29–31^. They also experience better mental health, with lower/reduced rates and severity of depression, hopelessness, and negative affect^25,32,33^, and also enjoy superior physical health, marked by reduced risk of cardiovascular disease, cognitive impairment, and mortality^28,32,34–36^.

Promising initial efforts aimed at enhancing purpose and meaning have been developed and evaluated, but they have generated inconsistent results^13,14^. A major challenge in crafting effective interventions is identifying definitive factors that enhance purpose and meaning. Research has explored various correlates and they can be categorized into: (1) psychological well-being, such as increased: positive affect, life satisfaction, optimism, and sense of control^37–40^, (2) psychological distress, including decreased: depressive symptoms, anxiety, negative affect, hopelessness, and early-life adversity/abuse^37–42^, (3) social factors, including increased: frequency of contact with friends, perceived social support, volunteering, social activities, religious service attendance, positive relationship with parents, as well as decreased loneliness and social exclusion^37,39,40,43–49^, (4) health factors, like maintaining: good health^37,50^ and physical activity^24,37^, (5) religion and spirituality, including: regular religious service attendance^37,38,43,51^, (6) socioeconomic factors, including: higher education, income, and wealth^52^. However, few studies have focused on the potential impact of childhood experiences on adult purpose and meaning. Existing research, mostly cross-sectional, has uncovered important insights, like how the absence of early-life adversity or abuse^41,42,53^, and positive relationships with parents during childhood^45,46^, are associated with increased purpose and meaning in adulthood. Without this knowledge, designing effective childhood interventions to foster purpose and meaning in adulthood remains challenging.

These prior studies have made critical contributions to the research literature, yet remain somewhat limited. First, there is a scarcity of research directly examining childhood correlates of adult purpose and meaning. Targeting health assets like purpose and meaning during childhood—a critical developmental phase for acquiring health assets and establishing healthy behaviors and mindsets—is a promising point of intervention that can substantially enhance the trajectory of health and well-being across the life course. Second, nearly all existing studies have been confined to single-country analyses, limiting our understanding of how different sociopolitical and economic contexts might shape purpose and meaning. Third, many studies did not adequately adjust for key potential confounders (e.g., some only adjusted for basic demographics). Fourth, most studies only evaluated a limited number of correlates, hindering our ability to compare effect sizes and identify the most impactful intervention targets.

In response to these identified gaps in the existing literature, our study aimed to address several of these limitations. Using data from a diverse, international sample of 202,898 people across 22 countries, our study aimed to answer three central questions: (#1) How do 11 aspects of a child’s upbringing correlate with sense of purpose and meaning in adulthood? (#2) Do these associations vary by country? (#3) Are the observed relationships robust to potential unmeasured confounding? This hypothesis-generating, data-driven approach allowed us to identify promising antecedents of purpose and meaning, which can then undergo further investigation in future studies.

## Results

Table 1 provides the distribution of descriptive statistics. Participant ages ranged from the entire adult lifespan (18–80+). The gender distribution was nearly balanced, with 51% female, 49% male, along with a small representation from other gender identities (<1%). Most participants were: married (53%), attained 9–15 years of education (57%), native-born (94%), and employed by an employer (39%). Regular attendance at religious services varied, with most never attending (37%), some attending once a week (19%), and others attending more than once a week (13%).

**Table 1:** Nationally-representative descriptive statistics of the observed sample (*N* = 202,898)

Variable	Proportion	Frequency
*Relationship with Mother*		
Very Good	63%	127,836
Somewhat Good	26%	52,439
Somewhat Bad	5%	11,060
Very Bad	2%	4642
Not Applicable	3%	5965
Missing	0%	956
*Relationship with Father*		
Very Good	53%	107,742
Somewhat Good	27%	55,714
Somewhat Bad	8%	15,807
Very Bad	4%	8278
Not Applicable	7%	13,985
Missing	1%	1372
*Parent Marital Status*		
Married	75%	152,001
Divorced	9%	17,726
Never Married	8%	15,534
One or Both Had Died	4%	7794
Missing	5%	9843
***Childhood Income***		
Lived Comfortably	35%	70,861
Got By	41%	82,905
Found it Difficult	18%	35,852
Found it Very Difficult	6%	12,606
Missing	0%	674
*Childhood Abuse*		
Yes	14%	29,139
No	82%	167,279
Missing	3%	6479
*Outsider*		
Yes	14%	28,732
No	84%	170,577
Not Applicable	1%	2712
Missing	0%	877
*Childhood Health*		
Excellent	33%	67,121
Very Good	31%	63,086
Good	23%	47,378
Fair	10%	19,877
Poor	2%	4906
Missing	0%	530
*Immigration Status*		
Born in This Country	94%	190,998
Born in Another Country	5%	9791
Missing	1%	2110
*Childhood Service Attendance*		
At Least 1/Week	41%	83,237
1–3/Month	16%	33,308
<1/Month	18%	36,928
Never	23%	47,445
Missing	1%	1980
*Gender*		
Male	49%	98,411
Female	51%	103,488
Other	0%	602
Missing	0%	397
*Year of Birth*		
1998–2005; Age 18-24	13%	27,007
1993–1998; Age 25-29	10%	20,700
1983–1993; Age 30-39	20%	40,256
1973–1983; Age 40-49	17%	34,464
1963–1973; Age 50-59	16%	31,793
1953–1963; Age 60-69	14%	27,763
1943–1953; Age 70-79	8%	16,776
1943 or Earlier; 80 or Older	2%	4119
Missing	0%	20
*Country*		
Argentina	3%	6724
Australia	2%	3844
Brazil	7%	13,204
Egypt	2%	4729
Germany	5%	9506
Hong Kong	1%	3012
India	6%	12,765
Indonesia	3%	6992
Israel	2%	3669
Japan	10%	20,543
Kenya	6%	11,389
Mexico	3%	5776
Nigeria	3%	6827
Philippines	3%	5292
Poland	5%	10,389
South Africa	1%	2651
Spain	3%	6290
Sweden	7%	15,068
Tanzania	4%	9075
Turkey	1%	1473
United Kingdom	3%	5368
United States	19%	38,312

^*^Country-specific descriptive statistics are available in the Online Supplement.

Table 2 shows associations between the 11 childhood candidate correlates and purpose and meaning in adulthood. Several candidate childhood factors were associated, on average across countries in the GFS, with an increased sense of purpose and meaning in adulthood, including: (1) experiencing excellent [*β* = 0.48, 95% CI: 0.31, 0.64] or very good health growing up [*β* = 0.24, 95% CI: 0.14, 0.33], (2) regular attendance at religious services at age 12, ranging from at least 1×/week [*β* = 0.38, 95% CI: 0.28, 0.49], to 1–3×/month [*β* = 0.26, 95% CI: 0.16, 0.37], less than 1×/month [*β* = 0.12, 95% CI: 0.05, 0.19], (3) very/somewhat good relationship with mother [*β* = 0.18, 95% CI: 0.10, 0.27], (4) very/somewhat good relationship father [*β* = 0.15, 95% CI: 0.07, 0.23], (5) living in a family that comfortably met its financial needs [*β* = 0.17, 95% CI: 0.09, 0.24], (6) and being a female [*β* = 0.10, 95% CI: 0.04, 0.16]. We also observed a gradual and positive association between age and levels of purpose and meaning, where *β* ranged from 0.06 for age 25–29 to 0.44 for age 80+, indicating a progressive increase in purpose and meaning with advancing age.

**Table 2:** Random effects meta-analysis of regression of purpose and meaning on childhood correlates

						Estimated proportion of effects by threshold			
Variable	Category	Est	95% CI	SE	<−0.10	>0.10	Heterogeneity (τ)	*I*^2^	Global *p*-value
Relationship with mother	(Ref: Very bad/somewhat bad)								<0.001**
	Very/somewhat good	0.18	(0.10, 0.27)	0.04	0.00	0.77	0.13	48.7	
Relationship with father	(Ref: Very bad/somewhat bad)								<0.001**
	Very/somewhat good	0.15	(0.07, 0.23)	0.04	0.09	0.64	0.14	65.2	
Parent marital status	(Ref: Parents married)								<0.001**
	No, divorced	−0.06	(−0.19, 0.06)	0.06	0.41	0.23	0.25	82.0	
	Single, never married	−0.08	(−0.21, 0.05)	0.07	0.36	0.23	0.25	79.6	
	No, one or both had died	−0.10	(−0.20, −0.01)	0.05	0.50	0.05	0.15	47.1	
Subjective financial status of the family growing up	(Ref: Got by)								<0.001**
	Lived comfortably	0.17	(0.09, 0.24)	0.04	0.05	0.68	0.16	85.0	
	Found it difficult	−0.05	(−0.10, 0.01)	0.03	0.32	0.09	0.09	53.7	
	Found it very difficult	−0.14	(−0.26, −0.01)	0.06	0.50	0.18	0.23	69.6	
Abuse	(Ref: No)								<0.001**
	Yes	−0.31	(−0.39, −0.23)	0.04	0.86	0.00	0.15	71.4	
Outsider growing up	(Ref: No)								<0.001**
	Yes	−0.29	(−0.38, −0.20)	0.05	0.82	0.00	0.19	77.4	
Self-rated health growing up	(Ref: Good)								<0.001**
	Excellent	0.48	(0.31, 0.64)	0.08	0.00	0.86	0.38	95.2	
	Very good	0.24	(0.14, 0.33)	0.05	0.00	0.64	0.20	86.9	
	Fair	−0.20	(−0.30, −0.10)	0.05	0.68	0.05	0.19	73.8	
	Poor	−0.18	(-0.38, 0.03)	0.11	0.59	0.23	0.41	75.6	
Immigration status	(Ref: Born in this country)								<0.001**
	No	0.04	(−0.11, 0.19)	0.08	0.32	0.41	0.28	75.8	
Age 12 religious service attendance	(Ref: Never)								<0.001**
	At least 1/week	0.38	(0.28, 0.49)	0.05	0.00	0.95	0.20	75.0	
	1–3/month	0.26	(0.16, 0.37)	0.06	0.00	0.82	0.22	77.8	
	Less than 1/month	0.12	(0.05, 0.19)	0.04	0.00	0.55	0.11	57.8	
Year of birth	(Ref: 1998–2005; age 18–24)								<0.001**
	1993–1998; age 25–29	0.06	(−0.02, 0.13)	0.04	0.14	0.36	0.14	60.2	
	1983–1993; age 30–39	0.17	(0.06, 0.29)	0.06	0.09	0.55	0.26	87.6	
	1973–1983; age 40–49	0.22	(0.05, 0.38)	0.08	0.18	0.68	0.38	92.8	
	1963–1973; age 50–59	0.28	(0.07, 0.49)	0.11	0.14	0.59	0.48	94.6	
	1953–1963; age 60–69	0.36	(0.13, 0.60)	0.12	0.18	0.77	0.54	94.5	
	1943–1953; age 70–79	0.41	(0.07, 0.74)	0.17	0.32	0.64	0.76	95.6	
	1943 or earlier; age 80+	0.44	(0.09, 0.79)	0.18	0.27	0.55	0.74	89.2	
Gender	(Ref: Male)								<0.001**
	Female	0.10	(0.04, 0.16)	0.03	0.05	0.45	0.14	85.7	
	Other	−0.50	(−0.85, −0.14)	0.18	0.83	0.17	0.62	78.0	

*Note*. *p < 0.05; **p < 0.004 (Bonferroni corrected threshold).

Several candidate childhood factors were associated, on average across countries in the GFS, with a decreased sense of purpose and meaning in adulthood, including: (1) experiencing abuse [*β* = −0.31, 95% CI: −0.39, −0.23], (2) feeling like an outsider growing up [*β* = −0.29, 95% CI: −0.38, −0.20], (3) experiencing fair health growing up [*β* = −0.20, 95% CI: −0.30, −0.10], (4) living in a family where it was very difficult to meet financial needs [*β* = −0.14, 95% CI: −0.26, −0.01], (5) one or both parents died [*β* = −0.10, 95% CI: −0.20, −0.01]. However, there was little evidence that, on average, immigration status in childhood or having parents who were divorced or never married were associated with subsequent purpose and meaning in adulthood.

The meta-analyses also revealed striking heterogeneity in how early-life conditions shape later-life purpose and meaning in adulthood across the 22 countries. For example, excellent self-rated health in childhood showed high heterogeneity (*τ* = 0.38, *I*² = 95.2%), indicating varied associations with adult purpose and meaning that potentially depend heavily on cultural and social contexts. Similarly, aspects of the family environment, such as parental marital status (with *τ* values 0.25 and *I*² levels reaching >80% for children from divorced or never-married families), exhibited considerable variability. This finding suggests that while the stability of the childhood home environment may influence adult purpose and meaning, the association differs across countries. The economic backdrop of one’s upbringing, represented by subjective financial status (*τ* = 0.23 for ‘found it very difficult,’ *I*² = 69.6%), also showed substantial heterogeneity across countries, highlighting the diverse impact of financial stability in childhood on later life purpose and meaning. Finally, birth year exhibited high heterogeneity, which increased with age (comparing 25–29 year-olds, *τ* = 0.14, *I*² = 60.2, while comparing them to 80+ year-olds, *τ* = 0.74, *I*² = 89.2).

Some childhood correlates showed a nearly universal association with adult purpose and meaning across countries. We observed nearly universal positive associations for: excellent or very good health growing up, and attending religious service at least once a week or 1–3×/month. We observed nearly universal negative associations for: abuse, and feeling like an outsider growing up. Other correlates varied more in strength and direction across countries. We observed generally positive associations with: relationship with mother, relationship with father, financially comfortable growing up, and attending religious services less than 1×/month, but effect sizes varied considerably. Conversely, other childhood correlates were mostly negative, including: experiencing a difficult or very difficult financial situation, experiencing fair self-rated health, or identifying as an Other for gender. Some correlates displayed mixed associations across countries, such as parents being divorced, single, or one or both parents having died, experiencing poor health growing up, being an immigrant, being female, birth cohort, and immigration status.

There were also interesting patterns and variations among childhood correlates. For example, the overall effect size across all countries for excellent, versus good, childhood health was 0.48 (95% CI: 0.31, 0.64) (Fig. S11) had effect sizes that were positive but varied substantially across countries. Countries with relatively advanced healthcare systems and lower childhood mortality rates, such as Hong Kong, Japan, and Sweden, showed larger effect sizes (Hong Kong: 1.57, 95% CI: 1.28, 1.86; Japan: 1.21, 95% CI: 1.10, 1.32; Sweden: 1.04, 95% CI: 0.92, 1.16). In contrast, countries facing greater public health challenges and higher childhood mortality rates showed effect sizes closer to zero (Nigeria: 0.00, 95% CI: −0.19, 0.20; Kenya: −0.03, 95% CI: −0.17, 0.10). Another factor that exhibited considerable heterogeneous effect sizes across countries is age/birth year (Figs. S21–27). In some countries (Japan, Israel, Egypt), the associations of age/birth year with purpose and meaning are negligible. In other countries (Sweden, the United States, Germany, Argentina, and Brazil), purpose and meaning increase steadily across age cohorts, while in a third group (including Tanzania, Kenya, and India), it is younger generations who report greater purpose and meaning.

Further, country-specific results are available in the Supplement (see Tables S1–S23c and Figs. S1–S27). Additionally, results disaggregated by purpose (see Tables S24–S47c and Figs. S28–S54) and meaning (see Tables S48–S71c and Figs. S55–S81) were largely similar to results from the main analyses that combined purpose and meaning together. However, there were some notable exceptions. For example, childhood correlates generally appeared to have stronger associations with meaning than with purpose. Specifically, experiencing the death of one or both parents, having poor self-rated health during childhood, and facing financial difficulties growing up had stronger associations with meaning compared to purpose. Additionally, factors such as living comfortably and being female appeared to have stronger positive associations with meaning than with purpose. However, these differences were relatively small.

For each childhood correlate, we calculated E-values to evaluate the sensitivity of results to unmeasured confounding. Our E-value analyses suggest that many observed associations were reasonably robust to unmeasured confounding (see Table 3). For instance, consider the association between the relationship with one’s mother and adult purpose/meaning. To fully explain away this association, an unmeasured confounder would need to be associated with both the mother–child relationship and purpose/meaning by ratios of at least 1.39 each, above and beyond the covariates we already controlled for. Additionally, to shift the confidence interval to include the null, an unmeasured confounder would need to be associated with both variables by a ratio of at least 1.26. Weaker associations from any unmeasured confounder would be insufficient to negate this observed association.

**Table 3:** Sensitivity of meta-analyzed childhood correlates to unmeasured confounding

Variable	Correlate (level)	E-value for Estimate	E-value for 95% CI
Relationship with mother	(Ref: Very bad/somewhat bad)		
	Very/somewhat good	1.39	1.26
Relationship with father	(Ref: Very bad/somewhat bad)		
	Very/somewhat good	1.34	1.21
Parent marital status	(Ref: Parents married)		
	No, divorced	1.20	1.00
	Single, never married	1.24	1.00
	No, one or both had died	1.27	1.05
Subjective financial status of family growing up	(Ref: Got by)		
	Lived comfortably	1.37	1.26
	Found it difficult	1.16	1.00
	Found it very difficult	1.32	1.08
Abuse	(Ref: No)		
	Yes	1.57	1.46
Outsider growing up	(Ref: No)		
	Yes	1.54	1.41
Self-rated health growing up	(Ref: Good)		
	Excellent	1.78	1.56
	Very good	1.47	1.33
	Fair	1.41	1.26
	Poor	1.38	1.00
Immigration status	(Ref: Born in this country)		
	No	1.16	1.00
Age 12 religious service attendance	(Ref: Never)		
	At least 1/week	1.66	1.53
	1–3/month	1.50	1.35
	Less than 1/month	1.30	1.18
Year of birth	(Ref: 1998–2005; age 18–24)		
	1993–1998; age 25–29	1.19	1.00
	1983–1993; age 30–39	1.38	1.19
	1973–1983; age 40–49	1.44	1.18
	1963–1973; age 50–59	1.53	1.22
	1953–1963; age 60–69	1.63	1.31
	1943–1953; age 70–79	1.69	1.21
	1943 or earlier; age 80+	1.73	1.25
Gender	(Ref: Male)		
	Female	1.26	1.14
	Other	1.81	1.33

## Discussion

We analyzed nationally representative data from 22 countries (*N* = 202,898) and evaluated the associations between 11 childhood candidate correlates and subsequent sense of purpose and meaning in adulthood. No single childhood correlate appeared to dominantly influence purpose and meaning in adulthood; instead, a combination of several childhood correlates appeared to be at play. Our findings converge with prior studies that identified the following correlates of increased purpose and meaning, including: good health^37,50^, absence of early-life adversity/abuse^41,42^, regular religious service attendance^44,45^, and positive relationships with parents^45,46^. Additionally, our observation that feeling like an outsider is associated with lower purpose and meaning aligns with similar findings from previous studies, which have shown that experiences of ‘being forgotten’ and social exclusion are linked with reduced levels of purpose and meaning^47,49^.

Some of our findings can be contextualized through the lens of contemporary studies that have theorized about the critical factors that help people cultivate a sense of purpose (and meaning in life)^54–59^. Although these theories vary in focus—sometimes focusing on specific populations such as adolescents, adults, or marginalized groups—and primarily address purpose rather than meaning, they collectively underscore five critical themes: (1) Nurturing interpersonal support networks, (2) health and well-being, (3) stable economic foundations, (4) Enriching cultural integration and identity, and (5) active community engagement.

Some of our findings concerning childhood social factors—such as (1) a good relationship with mother, (2) a good relationship with father, (3) experiencing abuse, (4) parental death, and (5) feeling like an outsider—might be best understood through the lens of ‘*Nurturing interpersonal support networks*,’ as well as Attachment Theory^60,61^. Early attachment experiences with primary caregivers serve as blueprints that shape our actions, thoughts, feelings, and how we relate to our inner selves, others, and the world around us. Secure attachments in childhood foster a coherent sense of self, positive self-image, and an environment of safety and exploration. All these factors allow children to engage the world with curiosity as well as confidence. This exploratory behavior accumulates into a rich and diverse range of experiences, knowledge acquisition, self-understanding, and mentalization (the process by which we interpret our own and others’ thoughts and feelings). In turn, these factors contribute to a robust and adaptable cognitive framework that enables children to eventually understand and imbue their lives with purpose and meaning. Conversely, insecure attachments—often influenced by experiences like abuse, neglect, or parental loss—can challenge early developmental trajectories and may contribute to a fragmented, negative self-view and a mistrustful worldview. These factors can make it more challenging for children to develop a strong sense of purpose and meaning over time.

Some of our findings around childhood health (experiencing excellent or very good health growing up) and childhood financial environment (living in a family that comfortably met its financial needs) align well with the concepts of ‘*Health and well-being*’ and ‘*Stable economic foundations*.’ These concepts can be contextualized through an extension of the Broaden-and-build theory, which suggests that positive emotions expand awareness and promote novel, exploratory thoughts and behaviors^62^. Good health and financial stability in childhood enhance a child’s capacity to participate in enriching activities that are laden with positive emotions. These enriching activities help children build skills, psychological resources, and behavioral repertoires that are essential for developing a sense of purpose and meaning in life. Conversely, experiences with poor childhood health and socioeconomic status are associated with heightened threat vigilance, mistrust, challenges in social relationships, impaired self-regulation, and unhealthy lifestyle choices^63^. These behaviors can increase the risk of psychological issues like depression and anxiety, and physical health problems like cardiovascular disease and higher mortality across the lifespan. Additionally, poor health and finances in childhood limit access to educational, vocational, volunteering, and other opportunities that serve as gateways to enriching activities. These challenges can collectively limit opportunities for children to engage in broadening and building activities, which in turn may reduce the ability to cultivate a robust sense of purpose and meaning.

Finally, our findings around regular religious service attendance during childhood likely stem, in part, from religions and spiritualities providing a comprehensive framework for making sense of the world and understanding one’s place in it. However, the contribution of childhood religious service attendance might also be further understood through the lens of ‘*Enriching cultural integration and identity*’ and ‘*Active community engagement*.’ These can be contextualized by role theory and the role accumulation hypothesis^64^, which suggests that roles within societal structures (e.g., for religious organizations, being a volunteer in community service projects, choir/musician member, youth group leader, holiday event organizer, etc.) can be powerfully influential^65^. These roles come with societal expectations and obligations that shape new behaviors and interactions, shaping how we learn to act, think, feel, and relate to others. Accumulation of, and engagement in, these role-identities provides a stable foundation and routines that embed us within their cultural and religious traditions, enhances identity formation, and also promotes participation in a community that supports moral and ethical development. Over time, these structured engagements may bolster a person’s ability to imbue life with a deeper sense of purpose and meaning. Similarly, participation in non-religious organizations (e.g., community service organizations, scouting groups, youth sports teams, or environmental clubs, music and arts clubs), might offer comparable roles that involve team cooperation, leadership development, and community service, which in turn contribute to the cultivation of purpose and meaning. These examples illustrate how structured community involvement, whether religious or secular, might provide the critical social infrastructure necessary for the development of purposeful and meaningful lives.

While early-life adversities can present challenges to developing purpose and meaning, there is potential for people to transform adverse situations into a strengthened sense of purpose and meaning. Some individuals report that reflecting on past experiences, whether positive or negative, helps them clarify goals and life direction^66^. Further, research suggests that the degree to which difficult past events (e.g., traumatic events) are viewed as central to one’s sense of self may influence whether individuals experience distress or growth following adversity^67^. This ‘reactive purpose’ pathway highlights how some individuals recalibrate life aims after adversity, integrating challenging experiences into their narrative to foster resilience and renew commitment to purpose- and meaning-driven behaviors^12,68,69^.

Our findings suggest that certain childhood factors may be particularly promising targets for intervention, pending validation through future research. For example, we found a strong association between excellent childhood health and adult purpose/meaning (*β* = 0.48). If future studies confirm this relationship is causal, practical interventions could include early wellness programs in schools promoting physical and mental health, along with ensuring access to regular health screenings and nutritious meals. Religious service attendance at age 12, especially at least once per week (*β* = 0.38), also demonstrated a strong association with adult purpose/meaning, underscoring the potential importance of structured, community-based engagement during formative years. Programs fostering community involvement could create partnerships with local community centers, civic organizations, or religious groups, encouraging children to engage in cooperative activities that embed them within supportive social structures, which also create a foundational sense of identity and belonging. In contrast, negative experiences, such as childhood abuse (*β* = −0.31) and feeling like an outsider (*β* = −0.29), also displayed noteworthy associations. School-based programs could provide teachers with training on recognizing signs of abuse or social exclusion. Counseling services could also be provided for children who experience such challenges, helping them to build coping skills. Positive relationships with parents, such as a very or somewhat good relationship with one’s mother (*β* = 0.18) or father (*β* = 0.15), also emerged as a correlate of later purpose and meaning. Interventions to strengthen family relationships could include parent–child communication workshops, offered at schools or community centers, which teach skills for open dialog and conflict resolution. Factors like socioeconomic status also appeared to play a role, but showed varied associations. For instance, “living comfortably” in terms of subjective financial status (*β* = 0.17) during childhood appears to foster greater purpose and meaning and financial hardship (e.g., “found it very difficult,” *β* = −0.14), linked to lower levels of purpose and meaning in adulthood. Programs designed to provide financial literacy and family support resources could help improve financial stability within families. Further, subsidized after-school programs could help children from all backgrounds have access to enriching activities. We encourage future research to continue exploring these types of ideas, which in turn can guide the development of targeted policies and interventions that support purpose and meaning development.

We also observed heterogeneity in cross-country associations. For instance, as Fig. S11 illustrates, excellent childhood health appears to more strongly be associated with subsequent purpose and meaning in countries which are relatively rich and (relatedly) have generally good healthcare systems and high life expectancy (cp. Hong Kong, Japan, Sweden, and the United States to Nigeria, Kenya, S. Africa, and India). This variation may partly stem from differences in public health conditions. In some countries, like Nigeria, there is a higher prevalence of health issues in children, such as malaria and malnutrition. While in others like, Japan, poor childhood health typically signals rare or severe ailments—which we would expect to be associated with less subsequent purpose and meaning.

Another childhood factor that exhibits considerable heterogeneous associations across countries is birth year (cf. Figs. S21–27). In some countries (Japan, Israel, Egypt), the associations of birth year with purpose and meaning are negligible. In others, such as Sweden, the United States, Germany, Argentina, and Brazil, purpose and meaning increase steadily across age cohorts, while in a third group (including Tanzania, Kenya, and India), it is younger generations who report greater purpose and meaning. We cannot determine from these data whether these associations are age-effects or cohort-effects, but there are indications that, at least in Western European and “Anglosphere” countries, overall adolescents’ flourishing—especially mental health—has been deteriorating at least since 2012^70^. A range of factors have been proposed for these trends, including the increasing substitution in these societies of a “play-based” for a “phone-based childhood”^70,71^. By contrast, rising levels of purpose and meaning by age in developing countries might reflect young people’s choices regarding education, profession, or other aspects of their life course.

This study has several limitations worth noting. First, all data for these analyses were collected at a single time point. Nonetheless, we aimed to create a retrospective longitudinal data structure, which allowed for a causal interpretation of the estimates. This approach is subject to unmeasured confounding, which we attempted to address by controlling for a range of potential confounders and formally evaluating the robustness of our findings using E-value analyses. These analyses suggest that many observed associations are moderately robust to unmeasured confounding. Additionally, while retrospective assessments can be influenced by recall bias, this bias would need to be strong to fully explain away the associations that we observed. Specifically, any bias in how adult levels of purpose and meaning impact memories of childhood experiences would need to be at least as strong as the observed associations themselves, many of which were quite substantial. Second, respondents in different countries might have interpreted the purpose and meaning items differently. However, analyses from cognitive interviews suggest that people were interpreting the items relatively similarly across countries^72^. Third, a key debate in the field is the content and contours of purpose in life vs. meaning in life vs. other related constructs. Growing theoretical and empirical work has helped define the contours of purpose and meaning as distinct constructs^2,6^. In our main results, we combine both purpose and meaning because it became unwieldy to interpret and discuss both constructs separately in this paper. However, we present disaggregated results looking at purpose and meaning separately in the Supplement. We encourage future researchers to evaluate how childhood factors might differentially influence purpose vs. meaning in adulthood. Fourth, our primary unit of analysis was the nation. This can be a problematic construct given the diversity within and across nations. For instance, Sweden is small, culturally homogenous, and has been relatively self-governing since the eleventh century, whereas Indonesia encompasses over 300 distinct languages and has been influenced by Buddhism, Hinduism, Islamism, Christianity, and Confucianism^73^. Fifth, we lacked a country-specific measure of socioeconomic status (SES) as a control variable. SES broadly influences a range of adult outcomes, so it is possible that its associations with well-being may partly underlie some associations observed between childhood correlates and adult purpose/meaning. Although we conducted all analyses by country, relevant indicators and the categorization of SES may differ across countries. Future research could clarify these relationships by incorporating SES measures tailored to each country. However, to partially address this limitation, we adjusted for subjective financial status during childhood, which captures a key aspect of SES. Future research should examine how socioeconomic contexts shape both the development and expression of purpose and meaning, as well as their correlates. Sixth, although purpose and meaning are complex concepts, each was assessed using a single-item measure. While this approach was necessary due to space constraints in a large cohort study, it has its limitations. For example, our purpose measure relied on a single-item question: “I understand my purpose in life.” While this item captures a person’s self-reported understanding of their purpose, it may not fully capture the complexity or depth of the construct. For example, understanding one’s purpose is an essential aspect, but it does not necessarily reflect the extent to which individuals are committed to actively pursuing that purpose. Future research should use multi-item scales that assess various dimensions of purpose (and meaning) in order to provide a more comprehensive and nuanced assessment.

This study also had notable strengths. It was among the first studies to evaluate childhood correlates of purpose and meaning, and also among the first studies to evaluate this question cross-nationally. Further, we used large, diverse, and nationally representative samples from 22 countries. We were also able to use well-thought-out questionnaires that were explicitly designed for the study of well-being, and not items that were repurposed from other studies originally created for different purposes.

These findings lay the groundwork for exciting avenues of future research. For example, future researchers may want to explore whether national-level characteristics, such as income inequality, religiosity, or cultural values (e.g., individualism-collectivism or ‘tightness-looseness’), influence how childhood experiences shape adult purpose and meaning. This is just a sampling of the potential areas to investigate; such research could help clarify the diverse sociocultural contexts that may amplify or dampen these associations.

Our findings highlight the potentially critical role that fostering robust early health, nurturing parental bonds, consistent communal or spiritual engagement, and financial stability play in helping individuals cultivate a heightened sense of purpose and meaning in adulthood. Conversely, our research also identified certain childhood adversities—such as experiencing abuse, feeling marginalized or like an outsider, encountering health challenges, enduring financial hardship, and coping with the loss of one or both parents—that were linked to diminished levels of purpose and meaning later in life. Additionally, our exploration into the heterogeneity of early-life conditions and their impact on adult purpose and meaning across 22 countries highlights the nuanced roles that health, family, and economic factors play in shaping adult well-being across nations. With further research, these findings could inform the development of globally adaptable, yet locally nuanced, programs and policies designed to foster purpose and meaning across the world^74^.

## Methods

The description of the methods below have been adapted from VanderWeele et al.^75^. Further methodological detail is available elsewhere^72,76–83^.

We used data from the Global Flourishing Study (GFS), which examines the distribution and determinants of well-being across a sample of 202,898 participants from 22 geographically and culturally diverse countries. Wave 1 of GFS collected nationally representative data from the following countries and territories: Argentina, Australia, Brazil, Egypt, Germany, Hong Kong, India, Indonesia, Israel, Japan, Kenya, Mexico, Nigeria, the Philippines, Poland, South Africa, Spain, Sweden, Tanzania, Türkiye, United Kingdom, and the United States. These countries were chosen to (1) maximize coverage of the world’s population, (2) ensure geographic, cultural, and religious diversity, and (3) prioritize feasibility and existing data collection infrastructure. Gallup Inc. conducted the data collection primarily in 2023, although some regions began in 2022; timing varied by country, and more information can be found elsewhere^79^. The precise sampling design to ensure nationally representative samples varied by country, and further details are available elsewhere^79^. The data are publicly available through the Center for Open Science (https://www.cos.io/gfs). The translation process followed the TRAPD model (translation, review, adjudication, pretesting, and documentation) for cross-cultural survey research (ccsg.isr.umich.edu/chapters/translation/overview). Further details about methodology and survey development are documented in the GFS Questionnaire Development Report^76^, GFS Methodology^79^, GFS Codebook^84^, and GFS Translations documents^85,86^. The Global Flourishing Study has been approved by several ethics committees, including the Harvard University IRB. Further, informed consent was obtained from all respondents.

### Purpose and meaning in life

Purpose was assessed by asking, “I understand my purpose in life.” Response options ranged from 0 = Strongly Disagree to 10 = Strongly Agree. Meaning was assessed by asking, “Overall, to what extent do you feel the things you do in your life are worthwhile?” Response options ranged from 0 = Not at All Worthwhile to 10 = Completely Worthwhile. A composite score was created by averaging responses to the purpose and meaning items above^87^.

Purpose in life and meaning in life are two terms that some studies have treated interchangeably, i.e., referring to the same underlying construct. However, conceptual and measurement literature has increasingly differentiated between these terms^1–3^. Each is considered to have a unique conceptual definition, antecedents, and outcomes. We recognize the conceptual differences in these constructs. However, because the number of studies considering the antecedents of either purpose in life or meaning in life is still relatively small, we do not differentiate them here. This is important future work that needs to be done, thus, we also present results that disaggregate the two indicators in the Supplement.

### Candidate childhood correlates

We evaluated 11 candidate correlates that were assessed at baseline. Each correlate is numbered below (#1-#11), and organized into five categories (A–E).

A. Demographic correlates, included: (#1) Age/Year of Birth (“What is your age? Please enter your age below.” Age was classified as 18–24, 25–29, 30–39, 40–49, 50–59, 60–69, 70–79, and 80+), (#2) Gender (“What is your gender?” Response options were: Male, Female, or Other), and (#3) Immigration status (“Were you born in this country, or not?” Response options were: Yes or No). Race/ethnicity was also assessed in most but not all countries, and by country-specific demographic categories that are locally meaningful (see GFS Codebook). Race/ethnicity was dichotomized for inclusion in the childhood correlate analyses within each country by using the most prevalent (plurality) group as the reference category. This factor was used as a control variable when available.
B. Socioeconomic correlates included: (#4) Subjective financial status of family growing up: (“Which one of these phrases comes closest to your own feelings about your family’s household income when you were growing up, such as when YOU were around 12 years old?” Response options were: Lived comfortably, Got by, Found it difficult, or Found it very difficult.
C. Family dynamics and relationship correlates, included: (#5) Parents marital status/Family structure (“Were your parents married to each other when YOU were around 12 years old?” Response options were: Married, Divorced, Never married, One or both had died), (#6) Relationship with mother (“Please think about your relationship with your mother when you were growing up. In general, would you say that relationship was…” Response options were: Very good, Somewhat good, Somewhat bad, or Very bad?” Responses were dichotomized to Very/Somewhat good versus Very/Somewhat bad, (#7) Relationship with father (An analogous question and answers were used to assess relationship with father). “Does not apply” was treated as a dichotomous control variable for respondents who did not have a mother or father due to death or absence, (#8) Felt like an outsider in family growing up (“When you were growing up, did you feel like an outsider in your family?” Response options were: Yes or No), and (#9) Abuse (“Were you ever physically or sexually abused when you were growing up?” Response options were: Yes or No).
D. Health and well-being correlates, included: (#10) Self-rated health growing up (“In general, how was your health when you were growing up?” Response options were: Excellent, Very good, Good, Fair, or Poor?”).
E. Religious and spiritual correlates, included: (#11) Age 12 religious service attendance (“How often did YOU attend religious services or worship at a temple, mosque, shrine, church, or other religious building when YOU were around 12 years old?” Response options were: At least once/week, One to three times/month, Less than once/month, or Never. Childhood religious tradition/affiliation had response categories of Christianity, Islam, Hinduism, Buddhism, Judaism, Sikhism, Baha’i, Jainism, Shinto, Taoism, Confucianism, Primal/Animist/Folk religion, Spiritism, African-Derived, some other religion, or no religion/atheist/agnostic; precise response categories varied by country^85^. The “No religion/Atheist/Agnostic” group was used as the reference group when at least 3% of the observed sample within the country endorsed this category; otherwise, the most prominent religious group was used as the reference category.

Descriptive statistics for the observed sample, weighted to be nationally representative within the country, were estimated for each childhood demographic category. Within each country, we fit a weighted linear regression model with complex survey-adjusted standard errors to assess the associations between composite purpose and meaning and all 11 aforementioned childhood correlate variables, simultaneously. Following these individual country analyses, we used a random effects meta-analytic approach to aggregate the regression coefficients across all 22 countries^88,89^. We also estimated confidence intervals, lower and upper limits, 95% prediction intervals, heterogeneity (*τ*), and *I*^2^ for evidence concerning variation within a given demographic category across countries^90^. Forest plots of estimates are available in the online supplement. Religious affiliation/tradition and race/ethnicity were used within the country as control variables, when available, but these coefficients themselves were not included in the meta-analyses because categories/responses varied by country. All meta-analyses were conducted in R (R Core Team, 2024) using the metafor package^91^. Within each country, a global test of association of each childhood correlate variable group with outcome was conducted, and a pooled *p*-value^92^ across countries reported concerning evidence for association within any country. Bonferroni corrected p-value thresholds are provided based on the number of childhood demographic variables^93,94^. For each childhood correlate, we calculated E-values to evaluate the sensitivity of results to unmeasured confounding. An E-value is the minimum strength of the association an unmeasured confounder must have with both the outcome and the correlate, above and beyond all measured covariates, for an unmeasured confounder to explain away an association^95^. As a supplementary analysis, population-weighted meta-analyses of the regression coefficients were estimated. Further, we conducted analyses that evaluated purpose and meaning separately. All analyses were pre-registered with the Center for Open Science prior to data access, with only slight subsequent modification in the regression analyses due to multicollinearity https://osf.io/jc2mr. All code to reproduce analyses is openly available in an online repository^77^.

Missing data on all variables was imputed using multivariate imputation by chained equations, and five imputed datasets were used^96–98^. To account for variation in the assessment of certain variables across countries (e.g., religious affiliation/tradition and race/ethnicity), the imputation process was conducted separately in each country. This within-country imputation approach ensured that the imputation models accurately reflected country-specific contexts and assessment methods. Sampling weights were included in the imputation model to account for missingness, which was related to the probability of inclusion.

The GFS used different sampling schemes across countries based on the availability of existing panels and recruitment needs^79^. All analyses accounted for the complex survey design components by including weights, primary sampling units, and strata. Additional methodological detail, including accounting for the complex sampling design, is provided elsewhere^78^.

## Supplementary information

Supplementary Tables and Figures

## Data Availability

Data are available for download at https://www.cos.io/gfs.

## Code availability

All analyses were pre-registered with COS prior to data access (https://osf.io/jc2mr); all code to reproduce analyses is openly available in the online OSF repository.
